# Cigarette smoking and telomere length: A systematic review of 84 studies and meta-analysis

**DOI:** 10.1016/j.envres.2017.06.038

**Published:** 2017-10

**Authors:** Yuliana Astuti, Ardyan Wardhana, Johnathan Watkins, Wahyu Wulaningsih, for the PILAR Research Network

**Affiliations:** a Department of Surgery and Cancer, Imperial College London, London, UK; b Department of Obstetrics/Gynaecology, Faculty of Medicine, Universitas Gadjah Mada, Yogyakarta, Indonesia; c PILAR Research and Education, Cambridge, UK; d Department of Anaesthesiology, Faculty of Medicine, Universitas Gadjah Mada, Yogyakarta, Indonesia; e MRC Unit for Lifelong Health and Ageing at University College London, London, UK; f Division of Haematology/Oncology, Faculty of Medicine, Universitas Gadjah Mada, Yogyakarta, Indonesia

**Keywords:** Smoking, Tobacco, Ageing, Telomere, Telomere length

## Abstract

**Background:**

Cigarette smoking is a risk factor for ageing-related disease, but its association with biological ageing, indicated by telomere length, is unclear.

**Methods:**

We systematically reviewed evidence evaluating association between smoking status and telomere length. Searches were performed in MEDLINE (Ovid) and EMBASE (Ovid) databases, combining variation of keywords “smoking” and “telomere”. Data was extracted for study characteristics and estimates for association between smoking and telomere length. Quality of studies was assessed with a risk of bias score, and publication bias was assessed with a funnel plot. I^2^ test was used to observe heterogeneity. Meta-analysis was carried out to compare mean difference in telomere length by smoking status, and a dose-response approach was carried out for pack-years of smoking and telomere length. A sensitivity analysis was carried out to examine sources of heterogeneity.

**Results:**

A total of 84 studies were included in the review, and 30 among them were included in our meta-analysis. Potential bias was addressed in half of included studies, and there was little evidence of small study bias. Telomere length was shorter among ever smokers compared to never smokers (summary standard mean difference [SMD]: −0.11 (95% CI −0.16 to −0.07)). Similarly, shorter telomere length was found among smokers compared to non-smokers, and among current smokers compared to never or former smokers. Dose-response meta-analysis suggested an inverse trend between pack-years of smoking and telomere length. However, heterogeneity among some analyses was observed.

**Conclusion:**

Shorter telomeres among ever smokers compared to those who never smoked may imply mechanisms linking tobacco smoke exposure to ageing-related disease.

## Highlights

• Shorter telomere length is a marker for cellular ageing and chronic diseases.
• We systematically reviewed 84 primary studies on smoking and telomere length.
• Telomere length was shorter in ever smokers compared to never smokers.
• Among ever smokers, current smokers had shorter mean telomeres than former smokers.
• An inverse trend was indicated between pack-years of smoking and telomere length.

## Graphical abstract

**Figure:**
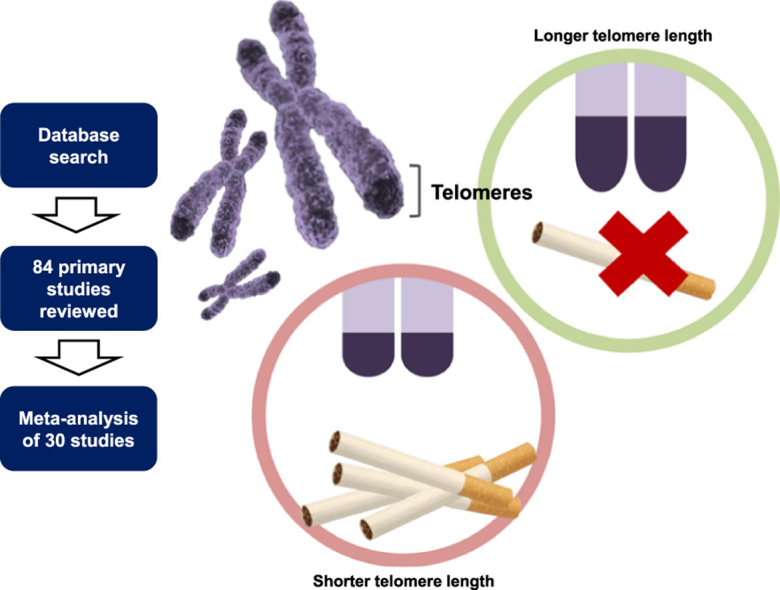

## 1 Introduction

Telomeres are ribonucleoprotein structures at the end of linear chromosomes essential for maintaining genome stability (Blackburn, 2001, Cech, 2008, O’Sullivan and Karlseder, 2010). Consisting of tandem arrays of TTAGGG sequence, telomeres serve as dispensable DNA sequences that shield genomic DNA from inevitable shortening during replication (Lingner et al., 1995). In addition, the special cap structure at the end of telomeric repeats, formed by 3’ G-strand overhang and telomere associated binding proteins, prevent recognition of the linear chromosome ends as DNA double strand break by the DNA repair machinery that may result in chromosome fusions (Griffith et al., 1999, Longhese, 2008, Van Steensel et al., 1998, Verdun and Karlseder, 2007).

Human telomeres shorten with each cell division and as telomeres become critically short, cells will cease proliferating and become senescent. As such, telomere length has long been considered as a marker of cellular aging (Bernadotte et al., 2016, Fyhrquist et al., 2011, Kuilman et al., 2010). In addition to genetic factor (Broer et al., 2013), environmental influences play an important role in determining telomere length (Huda et al., 2007, Starkweather et al., 2014). Tobacco smoking is a well-known health risk factor and exposure to harmful chemicals in cigarettes may induce oxidative stress and irreparable damage to the telomeric DNA (Alexandrov et al., 2006, Alexandrov et al., 2016, Asami et al., 1996; d’Adda di Fagagna et al., 2003; Opresko et al., 2005; Von Zglinicki, 2002). Despite this biological link, there have been inconsistencies in the literature regarding association between telomere length and smoking, with some studies showing shorter telomeres with smoking (Mirabello et al., 2009, Revesz et al., 2015) whereas a lack of association was reported in other studies (Brouilette et al., 2003, Harris et al., 2012). We therefore performed this systematic review and meta-analysis to determine whether combined evidence supports association between telomere length and smoking.

## 2 Methods

### 2.1 Search strategy

The meta-analysis was conducted according to the MOOSE (Stroup et al., 2000) And PRISMA guidelines (Moher et al., 2009). MEDLINE (Ovid) and EMBASE (Ovid) databases were searched from their inception to 29 April 2016, with the final search performed on 02/05/2016. We applied a search strategy as follows: (smoking OR cigarette*) AND (telomere OR telomeres) as free text. Searches were limited to studies conducted in humans. No language restriction was applied. References from eligible studies were hand-searched for additional studies. Two investigators independently identified eligible studies, and any discrepancies were resolved by consensus with a third investigator. There was no prior review protocol published for this study.

### 2.2 Inclusion criteria

We included studies that investigated an association between cigarette smoking status (including smoking status e.g. smokers, former smokers and never smokers and smoking intensity) and telomere length in humans, in which smoking status and telomere length were measured in the same subjects. Studies were either cross-sectional, cohort, or case-control studies in humans. We included studies in which smoking or telomere length was used as an adjustment variable if individual estimates of association between smoking and telomere length were available.

### 2.3 Exclusion criteria

Duplicated publications or additional studies of already included studies were excluded. We also excluded studies which did not fulfil any inclusion criteria, for instance, those which did not provide estimates for association between smoking and telomere length.

### 2.4 Data extraction

Data from eligible studies were independently extracted using a standard form. The following information was collected: first author, year of publication, type of study, description of study population (age, sex, race, country of study), method of telomere length measurement, source of sample used, description of smoking exposure assessment, sample size, comparison method, main results including maximally adjusted effect size and standard error or confidence intervals, any adjustment variables, and any other relevant information.

When information was available in the included studies, estimates for the following comparisons were collected: 1) current smokers and non-smokers, the latter of which consisted of former and never smokers, 2) ever smokers, which included both current and former smokers, and never smokers, 3) current smokers and former smokers, 3) current smokers and never smokers, 4) former smokers and never smokers, 5) smoking intensity, expressed as pack-years of cigarette, defined a product of packs of cigarettes smoked per day and smoking duration in years (Müezzinler et al., 2015), 6) levels of cotinine, a metabolite of nicotine (Block et al., 2006). When multiple measurements were available, we collected smoking status and telomere length measured at the same time, or closest to each other.

For studies which only reported estimates for categories e.g. quartiles of pack-year of cigarettes, we assigned interval scores of categories from the original studies based on medians or means when available. Category midranges were applied for the remaining closed-ended categories. For upper open-ended categories with *b*_*i*_ as the lower bound of the *i*th interval and the intervals indexed by *i* = 1,…,n, interval scores were assigned as *b*_*n*_ + 0.5 (*b*_*n*_ - *b*_*n-1*_) (Greenland and Longnecker, 1992, Il’yasova et al., 2005). Correspondingly, interval scores for the lower open-ended categories were assigned as *b*_*2*_ − 0.5 (*b*_*2*_ - *b*_*1*_).

### 2.5 Assessment of quality of included studies

Although quantitative scores have been used for meta-analysis of observational studies (Mundstock et al., 2015), interpretation could be challenging. We adapted assessment criteria from items in Critical Appraisal Skills Programme (CASP) questionnaires (Critical Appraisal Skills Programme, 2017) to assess cohort and case-control studies and use these criteria to assess included studies: (i) Did the study address a clearly focused issue? (ii) Did the authors use an appropriate method to answer their question? (iii) Was the exposure accurately measured to minimise bias? (iv) Was the outcome accurately measured to minimise bias? (v) Have they taken account of important confounding factors in the design and/or analysis? (vi) Do the results fit with other available evidence? Each item was answered with ‘Yes’, ‘No’ or ‘Don’t know’, according to information presented in the publications.

### 2.6 Assessment of publication bias

Assessment for publication bias was carried out by assessing funnel plot asymmetry for comparisons including at least 10 studies (Sterne et al., 2011). Data points were derived from estimates and standard errors from individual studies in relation to the pooled estimate effect. Asymmetrical distribution of data points for smaller studies (those with wider standard errors) indicates small study effects, which may be caused by publication bias. In addition to visual inspection of the funnel plot, we also conducted Egger's test, which applies weighted linear regression analysis to test for funnel plot asymmetry. A p-value of <0.1 was considered to represent significant asymmetry (Egger et al., 1997). Where asymmetry was indicated, sensitivity analysis was performed to seek potential sources of asymmetry.

### 2.7 Assessment of heterogeneity

The studies were evaluated clinically and methodologically to assess if it was reasonable to consider combining data. Statistical heterogeneity was measured by the visual inspection of the forest plots and statistically through an assessment of homogeneity based on the Chi^2^ test (Higgins and Greenland, 2011). The I^2^ measurement was calculated as an indicator of the amount of statistical variation not attributable to sampling error. A value of more than 75% was considered to represent high heterogeneity (Higgins et al., 2003).

### 2.8 Meta-analysis

A random effects meta-analysis was performed to obtain pooled results for each aforementioned comparison. Because different methods were used to assess telomere length, a standardised mean difference (SMD) approach was applied in the analysis. Summary results were obtained from final values and their variance in individual studies. A sensitivity analysis was performed by excluding studies one at a time. Where difference in means were presented for categories of exposure, e.g. for pack-year of smoking, we performed a two-stage meta-analysis approach (Crippa and Orsini, 2016). First, a dose-response model was estimated within each study taking into account the covariance of SMD corresponding to each assigned interval score. In the second stage, study-specific estimates were combined using a multivariate random effects model. Linear and quadratic dose-response associations were assessed, and Aikake information criterion (AIC) values were used to compare model fit. Where necessary, we performed a sensitivity analysis excluding one study at a time to determine potential sources of any observed heterogeneity. Analyses were performed using the package *metafor* and *dosresmeta* in R version 3.3.2 (R Foundation for Statistical Computing, Vienna, Austria Foundation for R Foundation for Statistical Computing, Vienna, Austriatatistical Computing, Vienna, Austria) and OpenMetaAnalyst software (Wallace et al., 2012).

## 3 Results

**Fig. 1:**
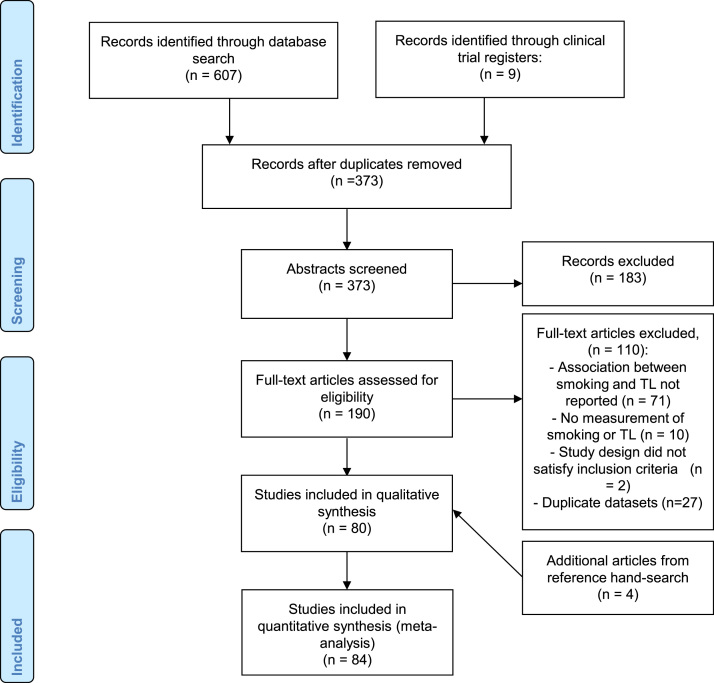
Selection of studies included in the systematic review.

As shown in Fig. 1, we identified a total of 616 studies from initial search, of which 373 study duplicates were removed. After title and abstract screening, full-text articles for 190 studies were retrieved. Among these articles, we further excluded 110 studies for reasons reported in Fig. 1 and full-text articles for the remaining 80 studies were retrieved for detailed reviewing. Finally, 4 additional studies identified among references in retrieved studies were included, resulting in a total of 84 articles selected for systematic review. Of these, 30 reported sufficient data for inclusion in meta-analysis.

### 3.1 Systematic review

Main characteristics of the included studies are summarised in Table 1, and references containing included studies are included in the Supplementary Information. Forty three studies were conducted in Europe, 25 in North America, 12 in Asia, 2 in Africa, and 2 in South America. In total, these studies involved 144,504 adults (age 18 and older) and most studies include both men and women (Table S1, Supplementary Information). Although some studies were longitudinal, all collected smoking status and telomere length were assessed cross-sectionally. Most studies used a single assessment of telomere length. Smoking status was self-reported in these studies. Measurements of telomere length were performed in the majority (75%) of the included studies and peripheral blood or leukocytes were mostly used as tissue source of DNA. Overall, among the 84 studies included, thirty-three reported shorter telomere length with smoking, one study reported longer telomere length with smoking, and fifty found a lack of association between smoking and telomere length. Only one included study (Verde et al., 2015) and a secondary analysis of study already included in the review (Patel et al., 2016) assessed the association between cotinine levels, in which shorter telomeres were indicated with higher cotinine levels. A meta-analysis was not possible because mean difference in telomere length was only available in one study. Time since quitting smoking was assessed in two studies (Müezzinler et al., 2015, Patel et al., 2016), and weak positive trends with telomere length were reported albeit different comparison methods hampered meta-analysis to be conducted.

**Table 1:** Description of studies included in the systematic review.

**First author, year**	**Country**	**No. of participants**	**Age**	**Telomere length assessment**	**Tissue source**	**Overall association**
Adams, 2007	UK	318	50	qPCR	PBMC	No association
Adler, 2013	USA	2599	70–79	qPCR	leukocytes	Shorter TL with smoking
Ahola, 2012	Finland	2911	30+	qPCR	leukocytes	No association
Aida, 2013	Japan	52	43–82	FISH	oesophageal tissue	No association
Ala-Mursula, 2013	Finland	5620	31	qPCR	leukocytes	No association
Atturu, 2010	UK	373	median 66	Southern blot	leukocytes	No association
Aulinas, 2015	Spain	154	mean 48.6	Southern blot	leukocytes	No association
Aviv, 2009	USA	635	24–44	Southern blot	blood	Shorter TL with smoking
Bakaysa, 2007	Sweden	175	mean 79	Southern blot	blood	No association
Balisteri, 2014	Italy	80	mean 63	Southern blot	blood	Shorter TL with smoking
Baragetti, 2015	Italy	768	mean 57	qPCR	leukocytes	Longer TL with smoking
Barcelo, 2010	Spain	404	46–52	qPCR	leukocytes	Shorter TL with smoking
Bekaert, 2007	Belgium	2509	35–55	Southern blot	leukocytes	No association
Bischoff, 2006	Denmark	812	73–101	Southern blot	leukocytes	No association
Boccardi, 2013	Italy	217	mean 78	qPCR	leukocytes	No association
Boyer, 2015	France	201	53–66	qPCR	blood	No association
Brody, 2014	USA	216	22	qPCR	PBMC*	No association
Brouilette, 2003	UK	203	mean 47	Southern blot	leukocytes	No association
Brouilette, 2007	UK	1542	45–64	qPCR	leukocytes	No association
Carty, 2016	USA	1525	50–79	Southern blot	leukocytes	Shorter TL with smoking
Cassidy, 2010	USA	2284	30–55	qPCR	leukocytes	No association
Chen, 2014	USA	3256	45–74	qPCR	blood	No association
Chen, 2015	Chile	89	18+	qPCR	saliva	Shorter TL with smoking
Demissie, 2006	USA	327	40–89	Southern blot	leukocytes	No association
Diez Roux, 2009	USA	1000	45–84	qPCR	blood	No association
Ehrlenbach, 2009	Austria	669	mean 62	qPCR	blood	No association
Fitzpatrick, 2007	USA	419	65+	Southern blot	leukocytes	No association
Fyhrquist, 2011	Finland	1271	55–80	Southern blot	leukocytes	No association
Gu, 2015	USA	1743	mean 78	qPCR	leukocytes	No association
Haque, 2013	UK	126	18+	qPCR	blood	Shorter TL with smoking
Harris, 2006	UK	190	79+	qPCR	leukocytes	No association
Harris, 2012	UK	1048	mean 69	qPCR	blood	No association
Haver, 2015	Netherland	3275	60+	qPCR	leukocytes	Shorter TL with smoking
Hou, 2009	Poland	716	21–79	qPCR	leukocytes	Shorter TL with smoking
Houben, 2009	Netherland	122	mean 63	qPCR	leukocytes	No association
Immonen, 2012	Finland	198	mean 75	Southern blot	blood	No association
Kahl, 2015	Brazil	124	17–68	qPCR	blood	Shorter TL with smoking
Kingma, 2012	Netherland	895	28–75	qPCR	leukocytes	No association
Kozlitina, 2012	USA	3157	18–85	qPCR	leukocytes	No association
Latifovic, 2016	Canada	477	20–50	qPCR	blood	No association
Lee, 2012	USA	4324	35–60	qPCR	leukocytes	No association
Lee, 2015	Asian	1958	40–69	qPCR	leukocytes	Shorter TL with smoking
Li, 2011	Sweden	166	19–65	qPCR	blood	No association
Lin, 2013	China	231	50–64	qPCR	oesophageal tissue	No association
Liu, 2015	Canada	922	19+	qPCR	leukocytes	Shorter TL with smoking
Liu, 2011	China	360	mean 50	qPCR	leukocytes	No association
Lynch, 2013	Finland	853	50–69	qPCR	blood	Shorter TL with smoking
Marcon, 2012	Italy	56	mean 56	Southern blot	blood	No association
Mirabello, 2009	USA	1661	55–74	qPCR	leukocytes	Shorter TL with smoking
Morla, 2006	Spain	76	55–62	FISH	leukocytes	Shorter TL with smoking
Muezzinler, 2015	Germany	3597	50–75	qPCR	leukocytes	Shorter TL with smoking
Nawrot, 2010	Belgium	305	mean 42.5	Southern blot	leukocytes	Shorter TL with smoking
Needham, 2013	USA	5360	20–84	qPCR	leukocytes	Shorter TL with smoking
Neuner, 2016	Germany	343	18–70	qPCR	blood	No association
Nordfjall, 2008	Sweden	989	26–75	qPCR	leukocytes	No association
Parks, 2009	USA	647	35–74	qPCR	blood	Shorter TL with smoking
Pavanello, 2011	Italy	457	25–75	qPCR	blood	Shorter TL with smoking
Pellatt, 2012	USA	1268	30–79	qPCR	blood	Shorter TL with smoking
Rane, 2015	Singapore	90	45–74	Southern blot	leukocytes	No association
Raymond, 2013	South Africa	450	case 51, control 40	qPCR	leukocytes	No association
Revesz, 2013	Netherland	2936	18–65	qPCR	leukocytes	Shorter TL with smoking
Risques, 2007	USA	300	30–89	qPCR	leukocytes	No association
Rode, 2014	Denmark	55,568	20–100	qPCR	leukocytes	Shorter TL with smoking
Sabatino, 2013	Italy	11	mean 67	qPCR	blood	Shorter TL with smoking
Sadr, 2015	Iran	189	mean 65	qPCR	blood	No association
Sanchez-Espiridion, 2014	USA	2790	mean 62	qPCR	lymphocytes	Shorter TL with smoking
Satoh, 1996	Japan	166	62–95	Southern blot	leukocytes	No association
Savale, 2009	France	291	55–70	qPCR	blood	No association
Song, 2010	USA	103	18–80	qPCR	T-lymphocytes	Shorter TL with smoking
Steptoe, 2011	UK	506	53–76	qPCR	PBMC*	No association
Strandberg, 2011	Finland	622	30–45	Southern blot	blood	Shorter TL with smoking
Surtees, 2012	UK	4441	41–80	qPCR	leukocytes	Shorter TL with smoking
Tsuji, 2006	Japan	34	mean 63	FISH	lung tissue	Shorter TL with smoking
Tyrka, 2015	USA	392	18–64	qPCR	blood	No association
Valdes, 2005	UK	561	mean 48.6	qPCR	leukocytes	Shorter TL with smoking
Verde, 2015	Spain	147	25–65	qPCR	leukocytes	No association
Von Kanel, 2015	South Africa	341	25–65	qPCR	leukocytes	No association
Wang, 2011	China	275	40–73	qPCR	leukocytes	No association
Wang, 2014	China	934	mean 43	qPCR	lymphocytes	Shorter TL with smoking
Whisman, 2016	USA	684	mean 53	qPCR	saliva	No association
Wong, 2014	USA	87	18+	qPCR	leukocytes	No association
Woo, 2009	China	4000	65+	qPCR	leukocytes	Shorter TL with smoking
Xiao, 2011	China	1797	mean 64	qPCR	leukocytes	Shorter TL with smoking
Zee, 2010	USA	518	40–84	qPCR	leukocytes	No association

Note: TL = telomere length; BMC = peripheral blood mononuclear cells; qPCR = quantitative polymerase chain reaction.

In addition to cross-sectional assessment of smoking status and telomere length, three studies had two repeated measures of telomere length. When adjusting for baseline telomere, telomere length measured 6 years later was inversely linked with number of cigarettes smoked at baseline (Kingma et al., 2012), but not smoking status (Aviv et al., 2009). Müezzinler and colleagues showed less telomere attrition over 8 years among current compared to never smokers (Müezzinler et al., 2015). Meta-analysis was not conducted due to the different types of measurements and interval time assessed.

### 3.2 Assessment of quality of included studies

To assess the risk of bias, we used criteria adapted from the CASP questionnaires for observational studies. As shown in Table S2 (Supplementary Information),all studies demonstrated clear aims, and adjustment for important confounding variables was applied in 42 out of 84 studies. Accurate measurements of exposure and outcome was indicated in the majority of studies, with seventy-six studies clearly described their methods in assessing LTL, and seventy one studies described how smoking was measured.

### 3.3 Assessment of publication bias

**Fig. 2:**
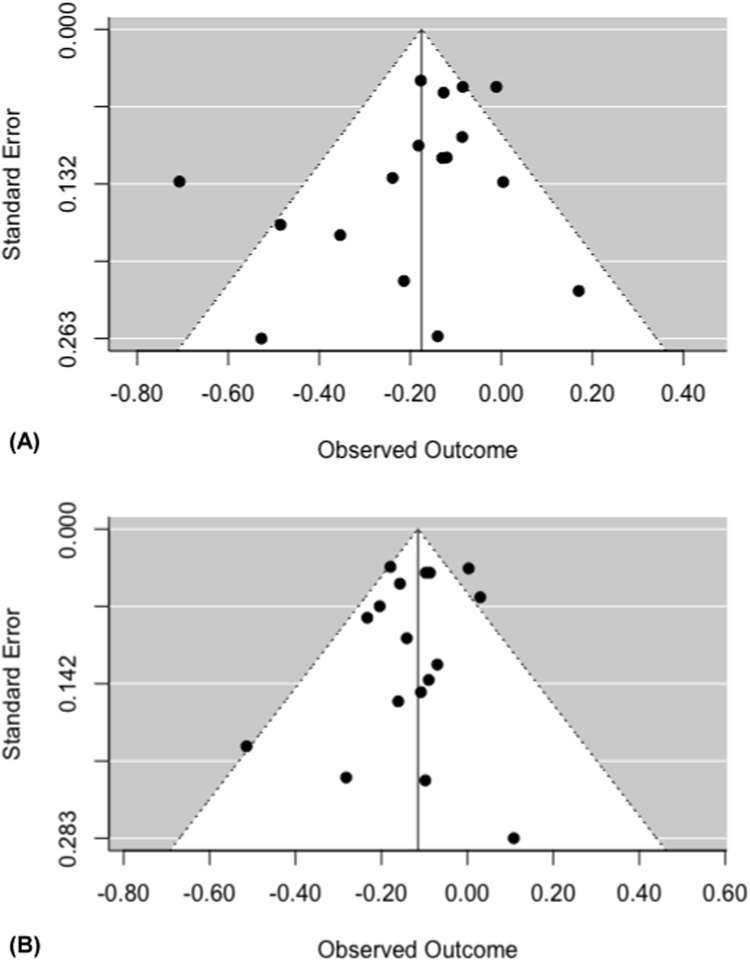
Funnel plots for associations between (A) smokers or non-smokers or (B) ever and never smokers and telomere length. X-axes represent standardized mean difference of telomere length and Y-axis represent their standard error.

Assessment of publication bias was conducted for the studies included in the meta-analysis which compared telomere length between smokers and non-smokers (Fig. 2A) as well as ever and never smokers (Fig. 2B), which each contained more than 10 studies. Both visual inspection and formal test for funnel plot asymmetry indicated symmetrical distribution (Egger's test p-values = 0.12 in analysis comparing smokers and non-smokers and 0.41 when comparing ever and never smokers), indicating that small study effects, which may reflect publication bias, were unlikely.

### 3.4 Meta-analysis of association between smoking and telomere length

**Fig. 3:**
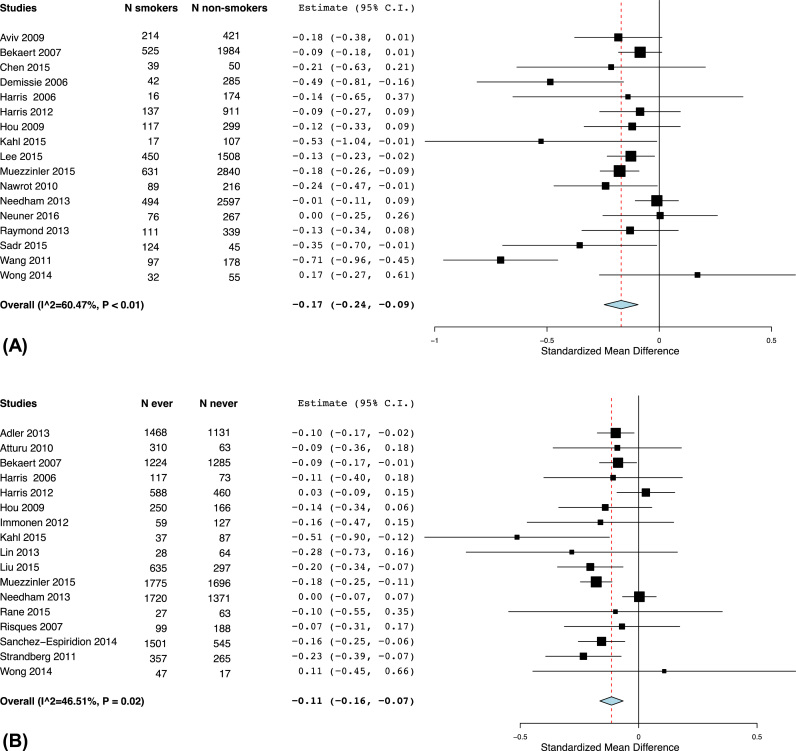
Comparison of means of telomere length between (A) smokers and non-smokers and (B) between ever or never smokers.

Meta-analyses were performed for 30 studies which provided comparable estimates for association between self-reported smoking exposures and telomere length. As several studies reported individual analyses either between smokers (current smokers) and non-smokers (former and never smokers) or ever (current and former smokers) smokers and never smokers, meta-analyses were carried out separately for each comparison. We found that telomere length were lower among smokers compared to non-smokers, with a pooled SMD of −0.17 (95% CI −0.24 to −0.09) (Fig. 3A). Moderate heterogeneity was found (I^2^ = 60.47%). Similarly, telomere length was lower among ever smokers than never smokers (Fig. 3B), with a summary SMD of −0.11 (95% CI −0.16 to −0.07), with I^2^ indicating a lack of substantial heterogeneity (I^2^ = 46.51%). A sensitivity analysis excluding one study at a time one at a time was performed in analysis comparing smokers and non-smokers. This analysis showed reduced heterogeneity (I^2^ = 26.87%) when a study by Wang and colleagues was removed, with summary SMD of −0.13 (95% CI −0.17 to −0.07) (Fig. S1).

**Fig. 4:**
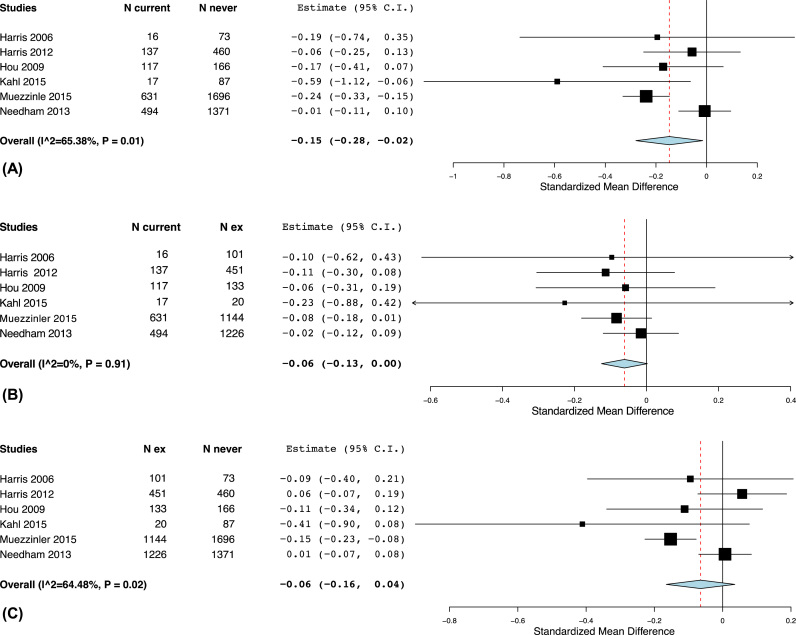
Comparison of means of telomere length between (A) current and former smokers, (B) between current and never smokers and (C) between former and never smokers.

We further evaluated difference in telomere length across current, former and never smokers. Telomere length was suggested to be lower among current compared to never smokers (summary SMD: −0.15, 95% CI: −0.28 to −0.02) and or compared to former smokers (summary SMD: −0.06, 95% CI: −0.13 to 0). No difference in mean telomere length was observed between former and never smokers (Fig. 4). There was moderate heterogeneity when comparing current to never smokers and high heterogeneity (I^2^ > 75%) when comparing former to never smokers. In the sensitivity analyses excluding one study at a time, we found that exclusion of a study by Needham et al. lowered the heterogeneity substantially with I^2^ less than 20% in comparison of telomere length between current and never smokers (Fig. S2, summary SMD: −0.20, 95% CI −0.30 to −0.10). Similar reduction in heterogeneity was shown by excluding a study by Muezzinler et al. when comparing former and never smokers (Fig. S3), with summary results indicating no difference in telomere length between the two groups (summary SMD: −0.01, 95% CI −0.09 to 0.07).

**Fig. 5:**
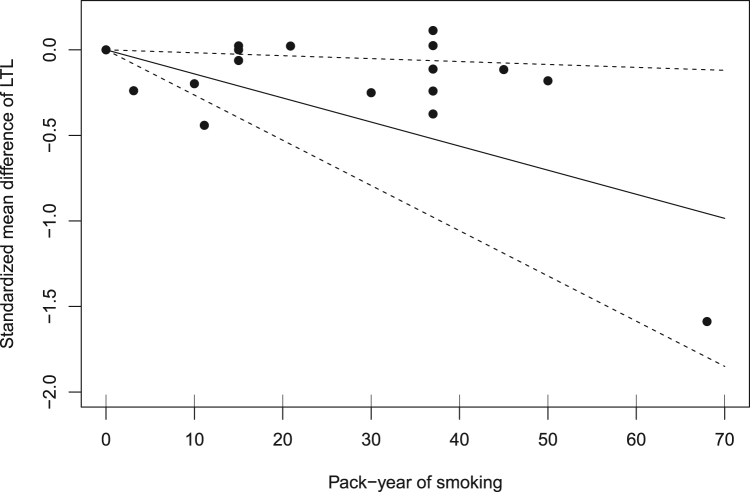
Dose-response association between pack-years of smoking and telomere length.

Five studies provided comparable estimates for mean difference of telomere length by categories of pack-years of smoking (Table S3, Supplementary Information), and they were included in a dose-response meta-analysis assessing the association between pack-year of smoking and telomere length. As shown in Fig. 5, assuming a linear relationship, there was an inverse trend between pack-years of smoking and telomere length. Each increase in pack-years of smoking corresponded to −0.01 decrease in mean telomere length (95% CI: −0.03 to −0.002) and high heterogeneity was indicated (I^2^ = 98.2%). Use of a quadratic model did not show any improvement of the model fit. In the sensitivity analysis, excluding any of the studies in the sensitivity analysis did not remove the observed heterogeneity.

## 4 Discussion

To our knowledge, this is the first systematic review and meta-analysis on cigarette smoking and telomere length. We found that telomere length was shorter among ever smokers compared to those never smoked. Among those who ever smoked, telomere length was indicated to be shorter among current smokers compared to those who quitted smoking. Using a dose-response meta-analysis, we found a weak inverse association between pack-years of smoking and telomere length. When assessing risk of bias, we found that half of included studies have adjusted for confounding variables in their analysis. Substantial heterogeneity was shown in some of the findings.

The observed associations may suggest that biological ageing, indicated by telomere length, is enhanced with active smoking. A plausible mechanism is that free radicals generated following cigarette smoking may induce oxidative stress and inflammation (Thorne et al., 2009), resulting in significantly shortened telomeres and lead to cellular senescence and apoptosis (d’Adda di Fagagna et al., 2003; Von Zglinicki, 2002). Our results also indicated the benefit of quitting smoking compared to being current smokers with regards to telomere length. There was a weak indication that former smokers who quitted smoking for a longer period of time may have longer telomeres compared to those who quit more recently or current smokers (Müezzinler et al., 2015, Wulaningsih et al., 2016), although a meta-analysis was not feasible. Additionally, higher pack-years of smoking, which reflect lifelong cigarette smoking exposure, corresponded to shorter telomeres although heterogeneity was shown. Altogether, these findings indicate the need to investigate whether there are cumulative effects of smoking on telomere length, and correspondingly, any reversal of such effects with longer period of smoking cessation. Studying the dynamic of telomere shortening may add understanding into such impact of smoking and cessation since it better reflects the biological impact of environmental influences compared to a single measurement of telomere length (Chen et al., 2011, Nordfjäll et al., 2009; Svenson et al., 2011). In the study by Müezzinler and colleagues in which repeated measures of telomere length 8-year apart were assessed, greater telomere loss was shown with longer period of quitting smoking among ever smokers, albeit non-significant (Müezzinler et al., 2015). The same study also showed that those who were current smokers at baseline had subsequent less telomere attrition than never smokers. Such conflicting finding may be explained by the “thrifty telomere” hypothesis (Eisenberg, 2011), which suggests that telomere length may act as a marker of one's limited resources over the life course or “disposable soma” (Kirkwood and Holliday, 1979). This theory states that having shorter telomeres to start with results in a more thrifty investment in maintenance efforts and reduced cell proliferation (Eisenberg, 2011), which may explain greater telomere length but more pronounced subsequent telomere loss with longer time of smoking cessation (Müezzinler et al., 2015). Further investigations regarding the role of smoking and its cessation on telomere length and attrition is therefore necessary to confirm such complex association.

The strength of this review lies in the use of different measures of smoking exposures ranging from self-report to objective biomarker measurements, and the inclusion of studies which included smoking as covariates when appropriate in addition to those evaluating smoking as the main exposure. We were able to estimate the dose-response relationship between smoking intensity, calculated as pack-years of smoking, with telomere length. Nevertheless, there was high heterogeneity when comparing telomere length between current and former smokers. Differences in population demographics may have explained such heterogeneity since exclusion of one Asian study (Wang et al., 2011) removed the heterogeneity observed when comparing ever and never smokers. However, less heterogeneity for other analyses were observed when excluding population-based studies which mostly included Caucasians (Müezzinler et al., 2015, Needham et al., 2013), which may indicate that there might have been factors other than ethnicities which explained the observed heterogeneity. It is unlikely that study design or telomere tissue explained this heterogeneity as all included studies in the analysis measured smoking status and telomere length cross-sectionally and used peripheral blood cells. Another possible source of heterogeneity might be the inter-laboratory technical variation of quantitative PCR (qPCR) to measure relative telomere length, which tend to yield higher variability than Southern blotting (Aviv et al., 2011, Gardner et al., 2014), although a study by Martin-Ruiz et al. suggested that both techniques show comparable intra- and inter-assay inconsistencies (Martin-Ruiz et al., 2015). Previous meta-analyses on cross-sectional associations between other lifestyle-related factors such as hypertension or obesity and leukocyte telomere length also reported high heterogeneity (Mundstock et al., 2015, Tellechea and Pirola, 2016), which may indicate the need to address methodological problem in telomere association study that should involve standardisation of qPCR and Southern blotting protocols (Martin-Ruiz et al., 2015).

Another limitation was that most included studies only used a single assessment of telomere length, which indicates the need to extend such analysis with repeated measurements. Furthermore, all studies included in the meta-analysis assessed circulating telomere length in blood. We were unable to assess any potential effect modification by sample source of telomere length such as saliva, which has also been linked to smoking (Zhang et al., 2016). Leukocyte telomere length has been widely used as a representative parameter of individual telomere status and that shorter leukocyte telomere length has been linked with increased mortality in several studies (Kimura et al., 2008, Needham et al., 2015). Nevertheless, the evidence regarding correlation of telomere length in leukocytes and other tissues is conflicting (Daniali et al., 2013, Dlouha et al., 2014, Friedrich et al., 2000, Gadalla et al., 2010, Lakowa et al., 2015, Mather et al., 2011). There are discrepancies in both telomere length and attrition rate of granulocytes and lymphocytes subpopulation (Mather et al., 2011, Sanders and Newman, 2013). Additionally, a study by Svenson et al. demonstrated fluctuation in leukocyte telomere length during the course of 6 months (Svenson et al., 2011). This fluctuation may be due to natural cycle or environment factors, such as acute infection, that changes the composition of leukocyte subpopulations and their replicative state (Akbar and Vukmanovic-Stejic, 2007, Hodes et al., 2002). Leukocyte is therefore not necessarily an ideal surrogate marker for other tissues in cross-sectional or short longitudinal studies. Most of our studies were of cross-sectional nature, therefore, our results only suggest association instead of causation. Having certain diseases, such as chronic obstructive lung disease, may result in smoking cessation and thus confound association between smoking status and telomere length. Estimates collected from included studies were adjusted for age, when information was available, in order to take into account the effect of age-related disease. However, residual confounding may still have occurred. We were able to capture lifelong exposure of smoking by pack-years of smoking, but this was based on self-report and therefore prone to recall bias. However, this was likely to result in non-differential misclassification because our outcome was a biomarker rather than any existing disease. Although we were unable to do a meta-analysis for cotinine levels, high agreement (~90%) has been shown between self-report active smoking status and cotinine levels (Arheart et al., 2008). To further tease out this association, a life course approach which allows assessment of smoking exposure prior to telomere length assessment, and use of repeated telomere measures may enhance understanding into the mechanisms of which smoking may influence telomere length.

## 5 Conclusion

In summary, this review showed that telomere length was shorter among ever smokers compared to those who never smoked, and among current smokers compared to former smokers. As shorter telomere may be a marker of early changes in ageing-related disease, the benefit suggested among former smokers compared to current smokers may further indicate the need to optimise policies targeting smoking cessation, particularly at earlier age. However, heterogeneity found in some of our findings indicates the need for further studies to quantify the implication of specific smoking exposures on telomere length and biological ageing.

## Conflict of interest

None declared

## Funding

WW is employed under grant from the UK Medical Research Council [MC_UU_12019/2; MC_UU_12019/4]. YA is supported by the Indonesian Endowment Fund for Education.

## Author Contributions

YA, AW and WW conceptualised the review. YA, AW and WW performed literature search, title and abstract screening, full text screening and data extraction. YA, JW and WW performed statistical analysis. YA and WW wrote the first draft of the manuscript. All authors provided feedback, reviewed and approved the final version of the manuscript.
