# Supplementary material for: Cigarette smoking and telomere length: A systematic review of 84 studies and meta-analysis
Source: Environ Res. 2017 Oct;158:480–9. doi: 10.1016/j.envres.2017.06.038 (PMC5562268; doi:10.1016/j.envres.2017.06.038)
Supplement: Supplementary file 1 — Supplementary material [file mmc1.docx]

**Table S1.** Summary of study population in included studies [1–84]

| Author | Year | Country | Age | Men/ women/ both | Race/ ethnicity | Total study size | Study name (if any) | Study design |
| --- | --- | --- | --- | --- | --- | --- | --- | --- |
| Adams | 2007 | UK | 50 | both | Not described | 318 | 1947 Newcastle Thousand Families Study | Cross sectional |
| Adler | 2013 | USA | 70-79 | both | Caucasian, African-American | 2599 | Health ABC | Cross sectional |
| Ahola | 2012 | Finland | 30+ | both | Not described | 2911 | Health 2000 Study | Cross sectional |
| Aida | 2013 | Japan | 43-82 | men | Asian | 52 |  | Cross sectional |
| Ala-Mursula | 2013 | Finland | 31 | both | Mostly Caucasian | 5620 | Northern Finland Birth Cohort 1966 | Cohort |
| Atturu | 2010 | UK | median 66 | both | Not described | 373 |  | Case control |
| Aulinas | 2015 | Spain | mean 48.6 | both | Not described | 154 |  | Cross sectional |
| Aviv | 2009 | USA | 24-44 | both | Caucasian, African-American | 635 | BHS | Cohort |
| Bakaysa | 2007 | Sweden | mean 79 | both | Not described | 175 | Swedish Twin Registry | Cohort |
| Balisteri | 2014 | Italy | mean 63 | both | Not described | 80 |  | Case control |
| Baragetti | 2015 | Italy | mean 57 | both | Not described | 768 | PLIC | Cross sectional |
| Barcelo | 2010 | Spain | 46-52 | both | Mostly Caucasian | 404 |  | Case control |
| Bekaert | 2007 | Belgium | 35-55 | both | Caucasian | 2509 |  | Cross sectional |
| Bischoff | 2006 | Denmark | 73-101 | both | Not described | 812 | LDCS | Cross sectional |
| Boccardi | 2013 | Italy | mean 78 | both | Caucasian | 217 |  | Cross sectional |
| Boyer | 2015 | France | 53-66 | both | Not described | 201 |  | Cross sectional |
| Brody | 2014 | USA | 22 | both | African-American | 216 |  | Cohort |
| Brouilette | 2003 | UK | mean 47 | both | Not described | 203 |  | Cross sectional |
| Brouilette | 2007 | UK | 45-64 | men | Not described | 1542 | WOSCOPS | Case control |
| Carty | 2016 | USA | 50-79 | both | Caucasian, African-American | 1525 | WHI | Cohort |
| Cassidy | 2010 | USA | 30-55 | women | Mostly Caucasian | 2284 | NHS | Nested case control |
| Chen | 2014 | USA | 45-74 | both | American-Indian | 3256 | SHSF | Cohort |
| Chen | 2015 | Chile | 18+ | both | Mostly Hispanic | 89 | CPASS | Cross sectional |
| Demissie | 2006 | USA | 40-89 | men | Caucasian | 327 | FHS | Cohort |
| Diez Roux | 2009 | USA | 45-84 | both | Caucasian, African-American, Hispanic | 1000 | MESA | Cohort |
| Ehrlenbach | 2009 | Austria | mean 62 | both | Mostly Caucasian | 669 | Bruneck Study | Cohort |
| Fitzpatrick | 2007 | USA | 65+ | both | Caucasian, African American | 419 | CHS | Cohort |
| Fyhrquist | 2011 | Finland | 55-80 | both | Not described | 1271 | LIFE | Cohort |
| Gu | 2015 | USA | mean 78 | both | Caucasian, Hispanic, African American, others | 1743 | WHICAP | Cohort |
| Haque | 2013 | UK | 18+ | women | Not described | 126 |  | Cross sectional |
| Harris | 2006 | UK | 79+ | both | Not described | 190 | LBC 1921 | Cohort |
| Harris | 2012 | UK | mean 69 | both | Caucasian | 1048 | LBC 1936 | Cohort |
| Haver | 2015 | Netherland | 60+ | both | Caucasian | 3275 | CORONA | RCT |
| Hou | 2009 | Poland | 21-79 | both | Not described | 716 |  | Case control |
| Houben | 2009 | Netherland | mean 63 | both | Not described | 122 |  | Case control |
| Immonen | 2012 | Finland | mean 75 | both | Not described | 198 |  | Case control |
| Kahl | 2015 | Brazil | 17-68 | both | Not described | 124 |  | Case control |
| Kingma | 2012 | Netherland | 28-75 | both | Not described | 895 | PREVEND | Cohort |
| Kozlitina | 2012 | USA | 18-85 | both | Caucasian, Hispanic, African American, others | 3157 | DHS | Cohort |
| Latifovic | 2016 | Canada | 20-50 | both | Not described | 477 |  | Nested case control |
| Lee | 2012 | USA | 35-60 | male | Mostly Caucasian | 4324 |  | Cohort |
| Lee | 2015 | Asian | 40-69 | both | Asian | 1958 | Korean Genome Epidemiology Study | Cohort |
| Li | 2011 | Sweden | 19-65 | both | Caucasian, Asian | 166 |  | Cross sectional |
| Lin | 2013 | China | 50-64 | both | Asian | 231 |  | Nested case control |
| Liu | 2015 | Canada | 19+ | both | Mostly Caucasian | 922 | CanCOLD | Cohort |
| Liu | 2011 | China | mean 50 | both | Asian | 360 |  | Case control |
| Lynch | 2013 | Finland | 50-69 | men | Mostly Caucasian | 853 | ATBC | Cohort |
| Marcon | 2012 | Italy | mean 56 | both | Not described | 56 |  | Cross sectional |
| Mirabello | 2009 | USA | 55-74 | men | Not described | 1661 | PLCO | Case control |
| Morla | 2006 | Spain | 55-62 | men | Not described | 76 |  | Cross sectional |
| Muezzinler | 2015 | Germany | 50-75 | both | Mostly Caucasian | 3597 | ESTHER | Cohort |
| Nawrot | 2010 | Belgium | mean 42.5 | both | Mostly Caucasian | 305 | FLEMENGHO | Cohort |
| Needham | 2013 | USA | 20-84 | both | Caucasian, Hispanic, African American, others | 5,360 | NHANES | Cross sectional |
| Neuner | 2016 | Germany | 18-70 | both | Mostly Caucasian | 343 |  | Cross sectional |
| Nordfjall | 2008 | Sweden | 26-75 | both | Mostly Caucasian | 989 | MDCC, MONICA | Cross sectional |
| Parks | 2009 | USA | 35-74 | women | Mostly Caucasian | 647 | The Sister Study | Cohort |
| Pavanello | 2011 | Italy | 25-75 | men | Caucasian | 457 |  | Case control |
| Pellatt | 2012 | USA | 30-79 | both | Mostly Caucasian | 1268 | DALS | Case control |
| Rane | 2015 | Singapore | 45-74 | both | Asian | 90 | SCHS | Cross sectional |
| Raymond | 2013 | South Africa | case 51, control 40 | both | African-American | 450 |  | Cross sectional |
| Revesz | 2016 | Netherland | 18-65 | both | Not described | 2936 | NESDA | Cohort |
| Risques | 2007 | USA | 30-89 | both | Not described | 300 | Seattle Barrett’s Esophagus Research Program | Cohort |
| Rode | 2014 | Denmark | 20-100 | both | Not described | 55,568 | CGPS | Cohort |
| Sabatino | 2013 | Italy | mean 67 | both | Not described | 11 |  | Cross sectional |
| Sadr | 2015 | Iran | mean 65 | both | Not described | 189 |  | Case control |
| Sanchez-Espiridion | 2014 | USA | mean 62 | both | Caucasian | 2790 |  | Case control |
| Satoh | 1996 | Japan | 62-95 | both | Asian | 166 |  | Cross sectional |
| Savale | 2009 | France | 55-70 | both | Mostly Caucasian | 291 |  | Case control |
| Song | 2010 | USA | 18-80 | both | Not described | 103 |  | Cross sectional |
| Steptoe | 2011 | UK | 53-76 | both | Caucasian | 506 | Whitehall II | Cohort |
| Strandberg | 2011 | Finland | 30-45 | men | Not described | 622 | HBS | Cohort |
| Surtees | 2012 | UK | 41-80 | both | Mostly Caucasio | 4441 | EPIC-Norfolk | Cohort |
| Tsuji | 2006 | Japan | mean 63 | both | Asian | 34 |  | Cross sectional |
| Tyrka | 2015 | USA | 18-64 | both | Caucasian, African-American, Hispanic, Asian, others | 392 |  | Cross sectional |
| Valdes | 2005 | UK | mean 48.6 | women | Not described | 561 | Twins UK | Cohort |
| Verde | 2015 | Spain | 25-65 | both | Not described | 147 |  | Cross sectional |
| Von Kanel | 2015 | South Africa | 25-65 | both | Caucasian, African-American | 341 | SABPA | Cross sectional |
| Wang | 2011 | China | 40-73 | men | Asian | 275 |  | Cross sectional |
| Wang | 2014 | China | mean 43 | both | Asian | 934 |  | Case control |
| Whisman | 2016 | USA | mean 53 | both | Caucasian, Hispanic, African American, others | 684 | HRS | Cohort |
| Wong | 2014 | USA | 18+ | men | Caucasian, African-American | 87 | Harvard Boilermakers | Cohort |
| Woo | 2009 | China | 65+ | both | Asian | 4000 |  | Cross sectional |
| Xiao | 2011 | China | mean 64 | both | Asian | 1797 |  | Case control |
| Zee | 2010 | USA | 40-84 | men | Not described | 518 | PHS | Case control |

**Table S2.** Risk of bias assessment

| Author | Year | Bias #1 - Validity of LTL measurement? | Bias #2 - Clear description of smoking assessment? | Bias #3 - Adequacy of statistical analysis techniques? | Bias #4 - Adjustment performed, at least for age? | Total bias score |
| --- | --- | --- | --- | --- | --- | --- |
| Adams | 2007 | 0.5 | 0.5 | 1 | 1 | 3 |
| Adler | 2013 | 1 | 0.5 | 1 | 1 | 3.5 |
| Ahola | 2012 | 1 | 0.5 | 1 | 0 | 2.5 |
| Aida | 2013 | 1 | 1 | 1 | 0 | 3 |
| Ala-Mursula | 2013 | 1 | 1 | 1 | 0 | 3 |
| Atturu | 2010 | 1 | 0.5 | 1 | 0 | 2.5 |
| Aulinas | 2015 | 0.5 | 0.5 | 1 | 0 | 2 |
| Aviv | 2009 | 1 | 1 | 1 | 1 | 4 |
| Bakaysa | 2007 | 1 | 0 | 1 | 0 | 2 |
| Balisteri | 2014 | 1 | 0 | 1 | 0 | 2 |
| Baragetti | 2015 | 1 | 0 | 1 | 0 | 2 |
| Barcelo | 2010 | 1 | 0 | 1 | 1 | 3 |
| Bekaert | 2007 | 1 | 0.5 | 1 | 1 | 3.5 |
| Bischoff | 2006 | 1 | 1 | 0 | 0 | 2 |
| Boccardi | 2013 | 1 | 0 | 1 | 0 | 2 |
| Boyer | 2015 | 0.5 | 0.5 | 0.5 | 1 | 2.5 |
| Brody | 2014 | 1 | 0.5 | 1 | 1 | 3.5 |
| Brouilette | 2003 | 1 | 0.5 | 1 | 1 | 3.5 |
| Brouilette | 2007 | 1 | 0 | 1 | 0 | 2 |
| Carty | 2016 | 1 | 0.5 | 1 | 1 | 3.5 |
| Cassidy | 2010 | 1 | 0.5 | 1 | 1 | 3.5 |
| Chen | 2014 | 1 | 1 | 1 | 1 | 4 |
| Chen | 2015 | 1 | 1 | 1 | 0 | 3 |
| Demissie | 2006 | 1 | 0 | 1 | 1 | 3 |
| Diez Roux | 2009 | 1 | 0.5 | 1 | 1 | 3.5 |
| Ehrlenbach | 2009 | 1 | 1 | 1 | 1 | 4 |
| Fitzpatrick | 2007 | 1 | 1 | 1 | 1 | 4 |
| Fyhrquist | 2011 | 1 | 0 | 0.5 | 0.5 | 2 |
| Gu | 2015 | 1 | 1 | 1 | 0 | 3 |
| Haque | 2013 | 1 | 0.5 | 1 | 1 | 3.5 |
| Harris | 2006 | 1 | 0 | 1 | 0 | 2 |
| Harris | 2012 | 1 | 0.5 | 1 | 1 | 3.5 |
| Haver | 2015 | 1 | 0.5 | 1 | 0.5 | 3 |
| Hou | 2009 | 1 | 0.5 | 1 | 1 | 3.5 |
| Houben | 2009 | 1 | 0 | 1 | 1 | 3 |
| Immonen | 2012 | 1 | 0.5 | 1 | 0 | 2.5 |
| Kahl | 2015 | 1 | 0.5 | 1 | 1 | 3.5 |
| Kingma | 2012 | 1 | 0.5 | 1 | 0 | 2.5 |
| Kozlitina | 2012 | 1 | 0.5 | 1 | 0.5 | 3 |
| Latifovic | 2016 | 1 | 1 | 1 | 1 | 4 |
| Lee | 2012 | 1 | 1 | 1 | 0 | 3 |
| Lee | 2015 | 1 | 0.5 | 1 | 0 | 2.5 |
| Li | 2011 | 1 | 0.5 | 0.5 | 1 | 3 |
| Lin | 2013 | 0.5 | 1 | 1 | 1 | 3.5 |
| Liu | 2015 | 1 | 0 | 1 | 1 | 3 |
| Liu | 2011 | 1 | 1 | 1 | 0 | 3 |
| Lynch | 2013 | 1 | 1 | 1 | 0 | 3 |
| Marcon | 2012 | 1 | 1 | 0 | 0 | 2 |
| Mirabello | 2009 | 1 | 0.5 | 1 | 1 | 3.5 |
| Morla | 2006 | 1 | 0.5 | 1 | 0 | 2.5 |
| Muezzinler | 2015 | 1 | 1 | 1 | 1 | 4 |
| Nawrot | 2010 | 1 | 1 | 1 | 1 | 4 |
| Needham | 2013 | 1 | 1 | 1 | 0 | 3 |
| Neuner | 2016 | 1 | 0.5 | 1 | 1 | 3.5 |
| Nordfjall | 2008 | 1 | 0 | 1 | 1 | 3 |
| Parks | 2009 | 1 | 0.5 | 1 | 1 | 3.5 |
| Pavanello | 2011 | 1 | 1 | 1 | 0 | 3 |
| Pellatt | 2012 | 1 | 0.5 | 1 | 0 | 2.5 |
| Rane | 2015 | 0.5 | 0.5 | 1 | 0 | 2 |
| Raymond | 2013 | 1 | 0 | 1 | 0 | 2 |
| Revesz | 2016 | 1 | 1 | 1 | 1 | 4 |
| Risques | 2007 | 1 | 0.5 | 1 | 1 | 3.5 |
| Rode | 2014 | 1 | 1 | 1 | 1 | 4 |
| Sabatino | 2013 | 1 | 0.5 | 1 | 0 | 2.5 |
| Sadr | 2015 | 1 | 0.5 | 1 | 0 | 2.5 |
| Sanchez-Espiridion | 2014 | 1 | 1 | 1 | 0 | 3 |
| Satoh | 1996 | 1 | 1 | 1 | 1 | 4 |
| Savale | 2009 | 0.5 | 1 | 1 | 0 | 2.5 |
| Song | 2010 | 1 | 1 | 1 | 0 | 3 |
| Steptoe | 2011 | 1 | 0 | 1 | 0 | 2 |
| Strandberg | 2011 | 1 | 0.5 | 1 | 1 | 3.5 |
| Surtees | 2012 | 0.5 | 0.5 | 1 | 1 | 3 |
| Tsuji | 2006 | 0.5 | 0.5 | 1 | 0 | 2 |
| Tyrka | 2015 | 1 | 0.5 | 0.5 | 0 | 2 |
| Valdes | 2005 | 1 | 1 | 1 | 1 | 4 |
| Verde | 2016 | 1 | 1 | 1 | 0 | 3 |
| Von Kanel | 2015 | 1 | 1 | 1 | 0.5 | 3.5 |
| Wang | 2011 | 1 | 0.5 | 0.5 | 1 | 3 |
| Wang | 2014 | 0.5 | 1 | 0.5 | 0 | 2 |
| Whisman | 2016 | 1 | 0 | 0.5 | 0.5 | 2 |
| Wong | 2014 | 1 | 0.5 | 1 | 0 | 2.5 |
| Woo | 2009 | 1 | 0.5 | 1 | 0 | 2.5 |
| Xiao | 2011 | 0.5 | 1 | 0.5 | 1 | 3 |
| Zee | 2010 | 1 | 0.5 | 1 | 1 | 3.5 |

**Table S3**. Studies included in the two-stage meta-analysis of association between pack-year of smoking and telomere length.

| Author, year | Pack-year of smoking^1^ | No. of participants | SMD of TL | Variance of SMD |
| --- | --- | --- | --- | --- |
| Needham, 2013 | 0 | 3055 | 0 | 0 |
|  | 15 | 1662 | 0.0231 | 0.0009 |
|  | 45 | 482 | -0.1155 | 0.0024 |
|  | 68 | 162 | -1.5881 | 0.0069 |
| Hou, 2009 | 0 | 166 | 0 | 0 |
|  | 15 | 137 | -0.0625 | 0.0133 |
|  | 37 | 112 | -0.375 | 0.0152 |
| Sanchez-Espiridion, 2014^2^ (a) | 15 | 192 | 0 | 0 |
|  | 37 | 386 | -0.2408 | 0.0078 |
| Sanchez-Espiridion, 2014^2^ (b) | 15 | 159 | 0 | 0 |
|  | 37 | 185 | 0.0246 | 0.0117 |
| Sanchez-Espiridion, 2014^2^ (c) | 15 | 41 | 0 | 0 |
|  | 37 | 270 | -0.1125 | 0.0281 |
| Sanchez-Espiridion, 2014^2^ (d) | 15 | 62 | 0 | 0 |
|  | 37 | 84 | 0.1128 | 0.0281 |
| Muezzinler, 2015 | 0 | 361 | 0 | 0 |
|  | 10 | 276 | -0.1982 | 0.0064 |
|  | 30 | 308 | -0.2507 | 0.0061 |
|  | 50 | 230 | -0.1809 | 0.0071 |
| Latifovic, 2016 | 0 | 328 | 0 | 0 |
|  | 3.125 | 66 | -0.2391 | 0.0183 |
|  | 11.125 | 43 | -0.4413 | 0.0266 |
|  | 20.875 | 42 | 0.022 | 0.0269 |

^1^Interval values are defined as explained in the Methods and previous studies [85, 86].

^2^Estimates were provided separately from different subgroups, indicated by (a), (b), (c), and (d).

TL=telomere length.


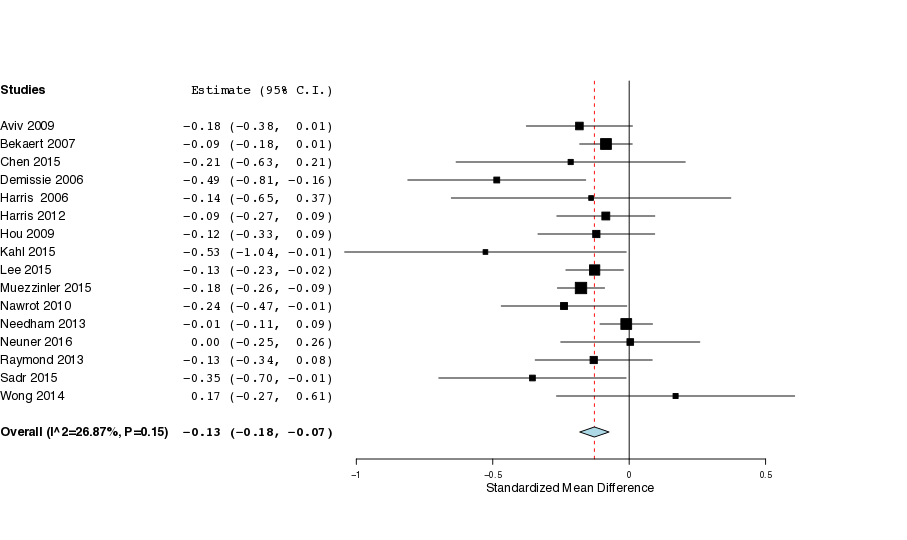
 **Figure S1.** Association between smokers or non-smokers and telomere length in the sensitivity analysis

**
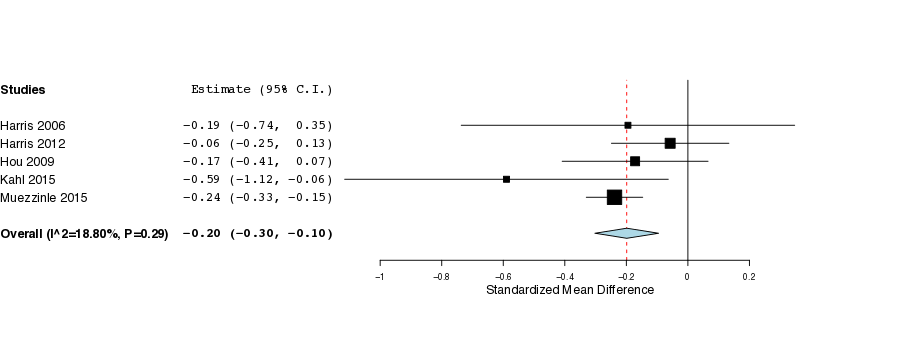
 Figure S2.** Association between current or never smokers and telomere length in the sensitivity analysis

**
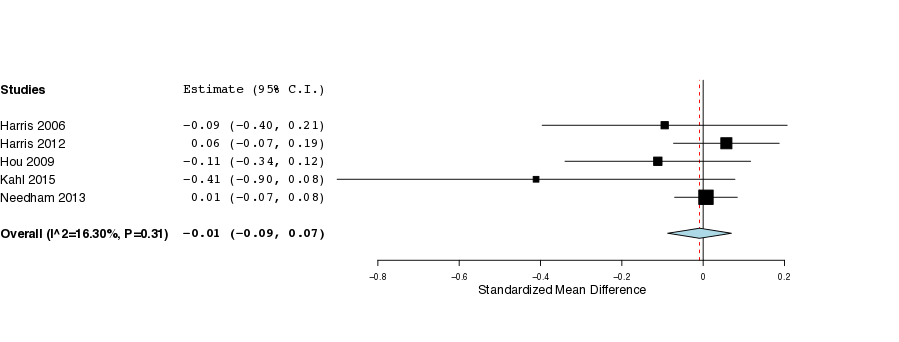
**

**Figure S3.** Association between former or never smokers and telomere length in the sensitivity analysis

**References**

1. Xie S-H, Liu A-L, Chen Y-Y, et al. (2010) DNA damage and oxidative stress in human liver cell L-02 caused by surface water extracts during drinking water treatment in a waterworks in China. Environ Mol Mutagen 51:229–235. doi: 10.1002/em

2. Adams J, Martin-Ruiz C, Pearce MS, et al. (2007) No association between socio-economic status and white blood cell telomere length. Aging Cell 6:125–128. doi: 10.1111/j.1474-9726.2006.00258.x

3. Adler N, Pantell MS, O’Donovan A, et al. (2013) Educational attainment and late life telomere length in the Health, Aging and Body Composition Study. Brain Behav Immun 27:15–21. doi: 10.1016/j.bbi.2012.08.014

4. Ahola K, Sirén I, Kivimäki M, et al. (2012) Work-related exhaustion and telomere length: A population-based study. PLoS One 7:13–15. doi: 10.1371/journal.pone.0040186

5. Aida J, Yokoyama A, Shimomura N, et al. (2013) Telomere Shortening in the Esophagus of Japanese Alcoholics: Relationships with Chromoendoscopic Findings, ALDH2 and ADH1B Genotypes and Smoking History. PLoS One 8:1–7. doi: 10.1371/journal.pone.0063860

6. Ala-Mursula L, Buxton JL, Ek E, et al. (2013) Long-term unemployment is associated with short telomeres in 31-year-old men: An observational study in the Northern Finland Birth Cohort 1966. PLoS One. doi: 10.1371/journal.pone.0080094

7. Atturu G, Brouilette S, Samani NJ, et al. (2010) Short Leukocyte Telomere Length is Associated with Abdominal Aortic Aneurysm (AAA). Eur J Vasc Endovasc Surg 39:559–564. doi: 10.1016/j.ejvs.2010.01.013

8. Aulinas A, Ramírez MJ, Barahona MJ, et al. (2015) Dyslipidemia and chronic inflammation markers are correlated with Telomere Length shortening in Cushing’s syndrome. PLoS One 10:1–15. doi: 10.1371/journal.pone.0120185

9. Aviv A, Chen W, Gardner JP, et al. (2009) Leukocyte telomere dynamics: Longitudinal findings among young adults in the Bogalusa Heart Study. Am J Epidemiol 169:323–329. doi: 10.1093/aje/kwn338

10. Bakaysa SL, Mucci LA, Slagboom PE, et al. (2007) Telomere length predicts survival independent of genetic influences. Aging Cell 6:769–774. doi: 10.1111/j.1474-9726.2007.00340.x

11. Balistreri CR, Pisano C, Martorana A, et al. (2014) Are the leukocyte telomere length attrition and telomerase activity alteration potential predictor biomarkers for sporadic TAA in aged individuals? Age (Omaha). doi: 10.1007/s11357-014-9700-x

12. Baragetti A, Palmen J, Garlaschelli K, et al. (2015) Telomere shortening over 6 years is associated with increased subclinical carotid vascular damage and worse cardiovascular prognosis in the general population. J Intern Med 277:478–487. doi: 10.1111/joim.12282

13. Barceló A, Piérola J, López-Escribano H, et al. (2010) Telomere shortening in sleep apnea syndrome. Respir Med 104:1225–1229. doi: 10.1016/j.rmed.2010.03.025

14. Bekaert S, De Meyer T, Rietzschel ER, et al. (2007) Telomere length and cardiovascular risk factors in a middle-aged population free of overt cardiovascular disease. Aging Cell 6:639–647. doi: 10.1111/j.1474-9726.2007.00321.x

15. Bischoff C, Petersen HC, Graakjaer J, et al. (2006) No Association Between Telomere Length and Survival Among the Elderly and Oldest Old. Epidemiology 17:190–194. doi: 10.1097/01.ede.0000199436.55248.10

16. Boccardi V, Esposito A, Rizzo MR, et al. (2013) Mediterranean Diet, Telomere Maintenance and Health Status among Elderly. PLoS One 8:4–9. doi: 10.1371/journal.pone.0062781

17. Boyer L, Chouaïd C, Bastuji-Garin S, et al. (2015) Aging-related systemic manifestations in COPD patients and cigarette smokers. PLoS One 10:1–12. doi: 10.1371/journal.pone.0121539

18. Brody GH, Yu T, Beach SRH, Philibert RA (2015) Prevention effects ameliorate the prospective association between nonsupportive parenting and diminished telomere length. Prev Sci 16:171–80. doi: 10.1007/s11121-014-0474-2

19. Brouilette SW, Moore JS, McMahon AD, et al. (2007) Telomere length, risk of coronary heart disease, and statin treatment in the West of Scotland Primary Prevention Study: a nested case-control study. Lancet 369:107–114. doi: 10.1016/S0140-6736(07)60071-3

20. Brouilette S, Singh RK, Thompson JR, et al. (2003) White cell telomere length and risk of premature myocardial infarction. Arterioscler Thromb Vasc Biol 23:842–846. doi: 10.1161/01.ATV.0000067426.96344.32

21. Carty CL, Kooperberg C, Liu J, et al. (2015) Leukocyte telomere length and risks of incident coronary heart disease and mortality in a racially diverse population of postmenopausal women. Arterioscler Thromb Vasc Biol 35:2225–2231. doi: 10.1161/ATVBAHA.115.305838

22. Cassidy A, De Vivo I, Liu Y, et al. (2010) Associations between diet, lifestyle factors, and telomere length in women. Am J Clin Nutr 91:1273–80. doi: 10.3945/ajcn.2009.28947

23. Chen S, Yeh F, Lin J, et al. (2014) Short leukocyte telomere length is associated with obesity in American Indians: the Strong Heart Family study. Aging (Albany NY) 6:380–9. doi: 10.18632/aging.100664

24. Chen X, Velez JC, Barbosa C, et al. (2015) Smoking and perceived stress in relation to short salivary telomere length among caregivers of children with disabilities. Stress 18:20–8. doi: 10.3109/10253890.2014.969704

25. Demissie S, Levy D, Benjamin EJ, et al. (2006) Insulin resistance, oxidative stress, hypertension, and leukocyte telomere length in men from the Framingham Heart Study. Aging Cell 5:325–330. doi: 10.1111/j.1474-9726.2006.00224.x

26. Diez Roux A V, Ranjit N, Jenny NS, et al. (2009) Race/ethnicity and telomere length in the Multi-Ethnic Study of Atherosclerosis. Aging Cell 8:251–7. doi: 10.1111/j.1474-9726.2009.00470.x

27. Ehrlenbach S, Willeit P, Kiechl S, et al. (2009) Influences on the reduction of relative telomere length over 10 years in the population-based bruneck study: Introduction of a well-controlled high-throughput assay. Int J Epidemiol 38:1725–1734. doi: 10.1093/ije/dyp273

28. Fitzpatrick AL, Kronmal RA, Gardner JP, et al. (2007) Leukocyte telomere length and cardiovascular disease in the cardiovascular health study. Am J Epidemiol 165:14–21. doi: 10.1093/aje/kwj346

29. Fyhrquist F, Silventoinen K, Saijonmaa O, et al. (2011) Telomere length and cardiovascular risk in hypertensive patients with left ventricular hypertrophy: the LIFE study. J Hum Hypertens 25:711–718. doi: 10.1038/jhh.2011.57

30. Gu Y, Honig LS, Schupf N, et al. (2015) Mediterranean diet and leukocyte telomere length in a multi-ethnic elderly population. Age (Omaha). doi: 10.1007/s11357-015-9758-0

31. Haque S, Rakieh C, Marriage F, et al. (2013) Shortened telomere length in patients with systemic lupus erythematosus. Arthritis Rheum 65:1319–23. doi: 10.1002/art.37895

32. Harris SE, Deary IJ, MacIntyre A, et al. (2006) The association between telomere length, physical health, cognitive ageing, and mortality in non-demented older people. Neurosci Lett 406:260–264. doi: 10.1016/j.neulet.2006.07.055

33. Harris SE, Martin-Ruiz C, von Zglinicki T, et al. (2012) Telomere length and aging biomarkers in 70-year-olds: The Lothian Birth Cohort 1936. Neurobiol Aging 33:1486.e3–1486.e8. doi: 10.1016/j.neurobiolaging.2010.11.013

34. Haver VG, Mateo Leach I, Kjekshus J, et al. (2015) Telomere length and outcomes in ischaemic heart failure: Data from the COntrolled ROsuvastatin multiNAtional Trial in Heart Failure (CORONA). Eur J Heart Fail 17:313–319. doi: 10.1002/ejhf.237

35. Hou L, Savage SA, Blaser MJ, et al. (2009) Telomere length in peripheral leukocyte DNA and gastric cancer risk. Cancer Epidemiol Biomarkers Prev 18:3103–3109. doi: 10.1158/1055-9965.EPI-09-0347

36. Houben JMJ, Mercken EM, Ketelslegers HB, et al. (2009) Telomere shortening in chronic obstructive pulmonary disease. Respir Med 103:230–236. doi: 10.1016/j.rmed.2008.09.003

37. Immonen I, Seitsonen S, Saionmaa O, Fyhrquist F (2013) Leucocyte telomere length in age-related macular degeneration. Acta Ophthalmol 91:453–456. doi: 10.1111/j.1755-3768.2012.02427.x

38. Kingma EM, de Jonge P, van der Harst P, et al. (2012) The Association between Intelligence and Telomere Length: A Longitudinal Population Based Study. PLoS One 7:1–6. doi: 10.1371/journal.pone.0049356

39. Kozlitina J, Garcia CK (2012) Red Blood Cell Size Is Inversely Associated with Leukocyte Telomere Length in a Large Multi-Ethnic Population. PLoS One 7:1–10. doi: 10.1371/journal.pone.0051046

40. Latifovic L, Peacock SD, Massey TE, King WD (2016) The Influence of Alcohol Consumption, Cigarette Smoking, and Physical Activity on Leukocyte Telomere Length. Cancer Epidemiol Biomarkers Prev 25:374–380. doi: 10.1158/1055-9965.EPI-14-1364

41. Lee J, Sandford AJ, Connett JE, et al. (2012) The Relationship between Telomere Length and Mortality in Chronic Obstructive Pulmonary Disease (COPD). PLoS One 7:e35567. doi: 10.1371/journal.pone.0035567

42. Lee J-Y, Jun N-R, Yoon D, et al. (2015) Association between dietary patterns in the remote past and telomere length. Eur J Clin Nutr 69:1048–1052. doi: 10.1038/ejcn.2015.58

43. Li H, J??nsson BAG, Lindh CH, et al. (2011) N-nitrosamines are associated with shorter telomere length. Scand J Work Environ Heal 37:316–324. doi: 10.5271/sjweh.3150

44. Lin S-W, Abnet CC, Freedman ND, et al. (2013) Measuring telomere length for the early detection of precursor lesions of esophageal squamous cell carcinoma. BMC Cancer 13:578. doi: 10.1186/1471-2407-13-578

45. Liu JCY, Leung JM, Ngan DA, et al. (2015) Absolute leukocyte telomere length in HIV-infected and uninfected individuals: Evidence of accelerated cell senescence in HIV-associated chronic obstructive pulmonary disease. PLoS One 10:1–13. doi: 10.1371/journal.pone.0124426

46. Liu J, Yang Y, Zhang H, et al. (2011) Longer leukocyte telomere length predicts increased risk of hepatitis b virus-related hepatocellular carcinoma: A case-control analysis. Cancer 117:4247–4256. doi: 10.1002/cncr.26015

47. Lynch SM, Major JM, Cawthon R, et al. (2013) A prospective analysis of telomere length and pancreatic cancer in the alpha-tocopherol beta-carotene cancer (ATBC) prevention study. Int J Cancer 133:2672–2680. doi: 10.1002/ijc.28272

48. Marcon F, Siniscalchi E, Crebelli R, et al. (2012) Diet-related telomere shortening and chromosome stability. Mutagenesis 27:49–57. doi: 10.1093/mutage/ger056

49. Mirabello L, Huang WY, Wong JYY, et al. (2009) The association between leukocyte telomere length and cigarette smoking, dietary and physical variables, and risk of prostate cancer. Aging Cell 8:405–413. doi: 10.1111/j.1474-9726.2009.00485.x

50. Morlá M, Busquets X, Pons J, et al. (2006) Telomere shortening in smokers with and without COPD. Eur Respir J 27:525–528. doi: 10.1183/09031936.06.00087005

51. Müezzinler A, Mons U, Dieffenbach AK, et al. (2015) Smoking habits and leukocyte telomere length dynamics among older adults: Results from the ESTHER cohort. Exp Gerontol 70:18–25. doi: 10.1016/j.exger.2015.07.002

52. Nawrot TS, Staessen JA, Holvoet P, et al. (2010) Telomere length and its associations with oxidized-LDL, carotid artery distensibility and smoking. Front Biosci (Elite Ed) 2:1164–8.

53. Needham BL, Adler N, Gregorich S, et al. (2013) Socioeconomic status, health behavior, and leukocyte telomere length in the National Health and Nutrition Examination Survey, 1999-2002. Soc Sci Med 85:1–8. doi: 10.1016/j.socscimed.2013.02.023

54. Neuner B, Lenfers A, Kelsch R, et al. (2015) Telomere length is not related to established cardiovascular risk factors but does correlate with red and white blood cell counts in a German blood donor population. PLoS One 10:1–13. doi: 10.1371/journal.pone.0139308

55. Nordfjäll K, Eliasson M, Stegmayr B, et al. (2008) Telomere Length Is Associated With Obesity Parameters but With a Gender Difference. Obesity 16:2682–2689. doi: 10.1038/oby.2008.413

56. Parks CG, Miller DB, McCanlies EC, et al. (2009) Telomere length, current perceived stress, and urinary stress hormones in women. Cancer Epidemiol Biomarkers Prev 18:551–560. doi: 10.1158/1055-9965.EPI-08-0614

57. Pavanello S, Hoxha M, Dioni L, et al. (2011) Shortened telomeres in individuals with abuse in alcohol consumption. Int J cancer 129:983–92. doi: 10.1002/ijc.25999

58. Pellatt AJ, Wolff RK, Lundgreen A, et al. (2012) Genetic and lifestyle influence on telomere length and subsequent risk of colon cancer in a case control study. Int J Mol Epidemiol Genet 3:184–94.

59. Rane G, Koh W-P, Kanchi MM, et al. (2015) Association between leukocyte telomere length and plasma homocysteine in a Singapore Chinese population. Rejuvenation Res 18:203–210. doi: 10.1089/rej.2014.1617

60. Raymond AR, Norton GR, Sareli P, et al. (2013) Relationship between average leucocyte telomere length and the presence or severity of idiopathic dilated cardiomyopathy in black Africans. Eur J Heart Fail 15:54–60. doi: 10.1093/eurjhf/hfs147

61. Révész D, Milaneschi Y, Terpstra EM, Penninx BWJH (2016) Baseline biopsychosocial determinants of telomere length and 6-year attrition rate. Psychoneuroendocrinology 67:153–162. doi: 10.1016/j.psyneuen.2016.02.007

62. Risques RA, Vaughan TL, Li X, et al. (2007) Leukocyte telomere length predicts cancer risk in Barrett’s esophagus. Cancer Epidemiol Biomarkers Prev 16:2649–2655. doi: 10.1158/1055-9965.EPI-07-0624

63. Rode L, Bojesen SE, Weischer M, Nordestgaard BG (2014) High tobacco consumption is causally associated with increased all-cause mortality in a general population sample of 55 568 individuals, but not with short telomeres: A Mendelian randomization study. Int J Epidemiol 43:1473–1483. doi: 10.1093/ije/dyu119

64. Sabatino L, Botto N, Borghini A, et al. (2013) Development of a new multiplex quantitative real-time PCR assay for the detection of the mtDNA(4977) deletion in coronary artery disease patients: a link with telomere shortening. Environ Mol Mutagen 54:299–307. doi: 10.1002/em.21783

65. Sadr M, Hossein SM, Mugahi N, et al. (2015) Telomere Shortening in Blood Leukocytes of Patients with Chronic Obstructive Pulmonary Disease. Tanaffos 14:10–16.

66. Sanchez-Espiridion B, Chen M, Chang JY, et al. (2014) Telomere length in peripheral blood leukocytes and lung cancer risk: A large case-control study in caucasians. Cancer Res 74:2476–2486. doi: 10.1158/0008-5472.CAN-13-2968

67. Satoh H, Hiyama K, Takeda M, et al. (1996) Telomere shortening in peripheral blood cells was related with aging but not with white blood cell count. Jpn J Hum Genet 41:413–417. doi: 10.1007/BF01876332

68. Savale L, Chaouat A, Bastuji-Garin S, et al. (2009) Shortened telomeres in circulating leukocytes of patients with chronic obstructive pulmonary disease. Am J Respir Crit Care Med 179:566–571. doi: 10.1164/rccm.200809-1398OC

69. Song Z, von Figura G, Liu Y, et al. (2010) Lifestyle impacts on the aging-associated expression of biomarkers of DNA damage and telomere dysfunction in human blood. Aging Cell 9:607–615. doi: 10.1111/j.1474-9726.2010.00583.x

70. Steptoe A, Hamer M, Butcher L, et al. (2011) Educational attainment but not measures of current socioeconomic circumstances are associated with leukocyte telomere length in healthy older men and women. Brain Behav Immun 25:1292–1298. doi: 10.1016/j.bbi.2011.04.010

71. Strandberg TE, Saijonmaa O, Tilvis RS, et al. (2011) Association of telomere length in older men with mortality and midlife body mass index and smoking. Journals Gerontol - Ser A Biol Sci Med Sci 66 A:815–820. doi: 10.1093/gerona/glr064

72. Surtees PG, Wainwright NWJ, Pooley KA, et al. (2012) Educational attainment and mean leukocyte telomere length in women in the European Prospective Investigation into Cancer (EPIC)-Norfolk population study. Brain Behav Immun 26:414–418. doi: 10.1016/j.bbi.2011.11.009

73. Tsuji T, Aoshiba K, Nagai A (2006) Alveolar cell senescence in patients with pulmonary emphysema. Am J Respir Crit Care Med 174:886–893. doi: 10.1164/rccm.200509-1374OC

74. Tyrka AR, Carpenter LL, Kao H-T, et al. (2015) Association of telomere length and mitochondrial DNA copy number in a community sample of healthy adults. Exp Gerontol 66:17–20. doi: 10.1016/j.exger.2015.04.002

75. Valdes AM, Andrew T, Gardner JP, et al. (2005) Obesity, cigarette smoking, and telomere length in women. Lancet 366:662–664. doi: 10.1016/S0140-6736(05)66630-5

76. Verde Z, Reinoso-Barbero L, Chicharro L, et al. (2015) Effects of cigarette smoking and nicotine metabolite ratio on leukocyte telomere length. Environ Res 140:488–94. doi: 10.1016/j.envres.2015.05.008

77. von Känel R, Malan NT, Hamer M, Malan L (2015) Comparison of Telomere Length in Black and White Teachers From South Africa. Psychosom Med 77:26–32. doi: 10.1097/PSY.0000000000000123

78. Wang S, Chen Y, Qu F, et al. (2014) Association between leukocyte telomere length and glioma risk: A case-control study. Neuro Oncol 16:505–512. doi: 10.1093/neuonc/not240

79. Wang Y-Y, Chen A-F, Wang H-Z, et al. (2011) Association of shorter mean telomere length with large artery stiffness in patients with coronary heart disease. Aging Male 14:27–32. doi: 10.3109/13685538.2010.529196

80. Whisman MA, Robustelli BL, Sbarra DA (2016) Marital disruption is associated with shorter salivary telomere length in a probability sample of older adults. Soc Sci Med 157:60–67. doi: 10.1016/j.socscimed.2016.03.029

81. Wong JYY, De Vivo I, Lin X, et al. (2014) The association between global DNA methylation and telomere length in a longitudinal study of boilermakers. Genet Epidemiol 38:254–64. doi: 10.1002/gepi.21796

82. Woo J, Suen EWC, Leung JCS, et al. (2009) Older men with higher self-rated socioeconomic status have shorter telomeres. Age Ageing 38:553–558. doi: 10.1093/ageing/afp098

83. Xiao F, Zheng X, Cui M, et al. (2011) Telomere dysfunction-related serological markers are associated with type 2 diabetes. Diabetes Care 34:2273–2278. doi: 10.2337/dc10-2431

84. Zee RYL, Castonguay AJ, Barton NS, Ridker PM (2010) Relative leukocyte telomere length and risk of incident ischemic stroke in men: a prospective, nested case-control approach. Rejuvenation Res 13:411–4. doi: 10.1089/rej.2009.0975

85. Il’yasova D, Hertz-Picciotto I, Peters U, et al. (2005) Choice of exposure scores for categorical regression in meta-analysis: A case study of a common problem. Cancer Causes Control 16:383–388. doi: 10.1007/s10552-004-5025-x

86. Greenland S, Longnecker MP (1992) Methods for trend estimation from summarized dose-response data, with applications to meta-analysis. AmJ Epidemiol 135:1301–1309. doi: 1626547
